# Clinical features of infectious endophthalmitis in South Korea: a five-year multicenter study

**DOI:** 10.1186/s12879-015-0900-5

**Published:** 2015-04-09

**Authors:** Ki Yup Nam, Joo Eun Lee, Ji Eun Lee, Woo Jin Jeung, Jung Min Park, Jong Moon Park, In Young Chung, Yong Seop Han, Il Han Yun, Hyun Wong Kim, Ik Soo Byon, Boo Sup Oum, Hee Sung Yoon, Dong Park, Byeng Chul Yu, Eun-Kee Park, Hu-Jang Lee, Sang Joon Lee

**Affiliations:** Department of Ophthalmology, College of Medicine, Kosin University, 262 Gamchun-ro, Seo-gu, Busan, South Korea; Department of Ophthalmology, College of Medicine, Inje University, Pusan, South Korea; Department of Ophthalmology, College of Medicine, Pusan National University, Pusan, South Korea; Department of Ophthalmology, College of Medicine, Dong-A University College of Medicine, Busan, South Korea; Department of Ophthalmology, Maryknoll Hospital, Busan, South Korea; Department of Ophthalmology, College of Medicine, Gyeongsang National University, Jinju, South Korea; Jung Geun Eye Hospital, Busan, South Korea; Sungmo Eye Hospital, Busan, South Korea; Su Jeong Eye Clinic, Busan, South Korea; Department of Preventive Medicine, College of Medicine, Kosin University, Pusan, South Korea; Department of Medical Humanities and Social Medicine, Kosin University, Pusan, South Korea; Research Institute of Life Sciences, College of Veterinary Medicine, Gyeongsang National University, Jinju, South Korea

**Keywords:** Endophthalmitis, Postoperative, Posttraumatic, Endogenous, Infection

## Abstract

**Background:**

To investigate clinical features of infectious endophthalmitis over five years in a South Korean population.

**Methods:**

Medical records of consecutive patients diagnosed with infectious endophthalmitis at eight institutions located in Gyeongsangnam-do and Pusan city between January 1, 2004 and July 31, 2010 were reviewed.

**Results:**

A total of 197 patients were diagnosed and treated. An average of 30.0 infectious endophthalmitis per year was developed. The annual incidence rate of postoperative endophthalmitis during 2006 ~ 2009 was 0.037%. The ratios of male to female and right to left were 50.2%: 49.8 % and 54.8%: 43.2%, respectively. Eighth decade and spring were the peak age (36.6%) and season (32.0%) to develop the infectious endophthalmitis. The most common past history in systemic disease was hypertension (40.4%), followed by diabetes (23.4%). Cataract operation (60.4%) was the most common cause, among which most of them was uneventful phacoemulsification (95.9%). Corneal laceration (51.6%) and liver abscess (42.9%) were the most common causes of traumatic and endogenous endophthalmitis, respectively. The percentages of patients with initial and final visual acuity less than counting fingers were 62.6% and 35.2%, respectively. Treatment with vitrectomy with or without intravitreal antibiotics injection was administered to 72.6% of patients, while 17.3% received intravitreal antibiotics only.

**Conclusions:**

Our study revealed that the development of infectious endophthalmitis was related with seasonal variation and increased during our study period. Pars plana vitrectomy was preferred for the treatment of infectious endophthalmitis in South Korea.

## Background

Infectious endophthalmitis, a serious intraocular inflammatory disorder that results from the exogenous or endogenous spread of infectious microorganisms into the eye [1], most commonly occurs after intraocular surgery but may also occur following ocular trauma or endogenous spread from adjacent periocular tissues or through the blood stream. A general trend towards a decreasing incidence of endophthalmitis has been observed in recent decades [2-4], with incidence rates ranging from 0.05 to 0.4% after intraocular surgery. However, endophthalmitis still accounts for significant visual morbidity [2]. Since the introduction of clear cornea incision techniques for cataract surgery, the rate of endophthalmitis has increased slightly [5].

Among several prominent endophthalmitis studies, the endophthalmitis vitrectomy study (EVS), a five-year, prospective, randomized, multicenter study, demonstrated the characteristics of postoperative infectious endophthalmitis in the USA. Recently, a multicenter infectious endophthalmitis epidemiologic study in Sweden was published [6]. Such studies can provide clinical features about the postoperative infectious endophthalmitis in those nations. Due to the low incidence of infectious endophthalmitis, single-center studies in South Korea had too few cases to reveal clinical characteristics. Despite this limitation, multiple papers have been published in Korea [7-10]. The conclusions of those articles insist that multicenter study of infectious endophthalmitis would be needed to overcome this weakness.

The present study included eight institutes based on their ability to perform pars plana vitrectomy and microbial culture and sensitivity testing to determine the clinical features and treatment pattern of infectious endophthalmitis over five years in South Korea population.

## Methods

This study was a retrospective, multicenter, consecutive longitudinal cross-sectional study based on the medical records of eight South Korean institutions from January 1, 2004 to July 31, 2010. Clinical and demographic data for all patients diagnosed by infectious endophthalmitis during that period were analyzed. The protocol of this study adhered to provisions of the Declaration of Helsinki, and was approved by the Institutional Review Board of Gyeongsang National University.

The following post-treatment data from ophthalmic centers at each institute were evaluated with the consensus protocol: case numbers of presumptive and culture-positive endophthalmitis; date of occurrence; sex; laterality; age; seasonal occurrence; past medical history; cause of infection (postoperative, posttraumatic, endogenous, or unspecified); duration of symptoms; interval between symptom occurrence and initial eye examination; preoperative visual acuity; final visual outcomes, which were defined as the best corrected vision achieved after at least one month of follow-up; treatment modality; topical, intravitreal, and systemic antibiotic selection; and culture positivity depending on the culture specimen and surgical tool. Visual acuity based on the Snellen chart was converted to log-MAR (log of the minimum angle of resolution) acuity for this analysis. For statistical analyses, Counting fingers was converted to 1.9 logMAR acuity. Hand movement, light perception and no light perception was converted to 2.3, 2.7, and 3.0, respectively [11].

The diagnosis of infectious endophthalmitis at each institute was based on patient’s clinical symptoms and signs. Presumptive endophthalmitis was confirmed by culture. Briefly, cultures were assessed from direct smears of aqueous or vitreous humor. Growth was tested on blood agar, chocolate agar, and thioglycolate broth. Cultures for fungi were performed at room temperature on Sabouraud agar without cycloheximide.

We also used data recorded between 2006 ~ 2009 in the national health claim database obtained from the Health Insurance Review and Assessment service of Korea (KIRA) to estimate the annual and monthly incidence of cataract operation [12]. The annual and seasonal incidences of postoperative endophthalmitis in Pusan and Gyungsangnam-do area of Korea were evaluated as follows. The number of postoperative infectious endophthalmitis in our study was divided by the number of cataract operation in the same district with our study. The Korea National Health Insurance scheme covers approximately 97% of the Korean population and is a compulsory social insurance [13]. The surgical codes for cataract operation are S5110 (pars plana lensectomy), S5111 (intracapsular cataract extraction), S5112 (aftercataract operation), and S5117 and S5119 (phacoemulsification).

## Results

**Figure 1:**
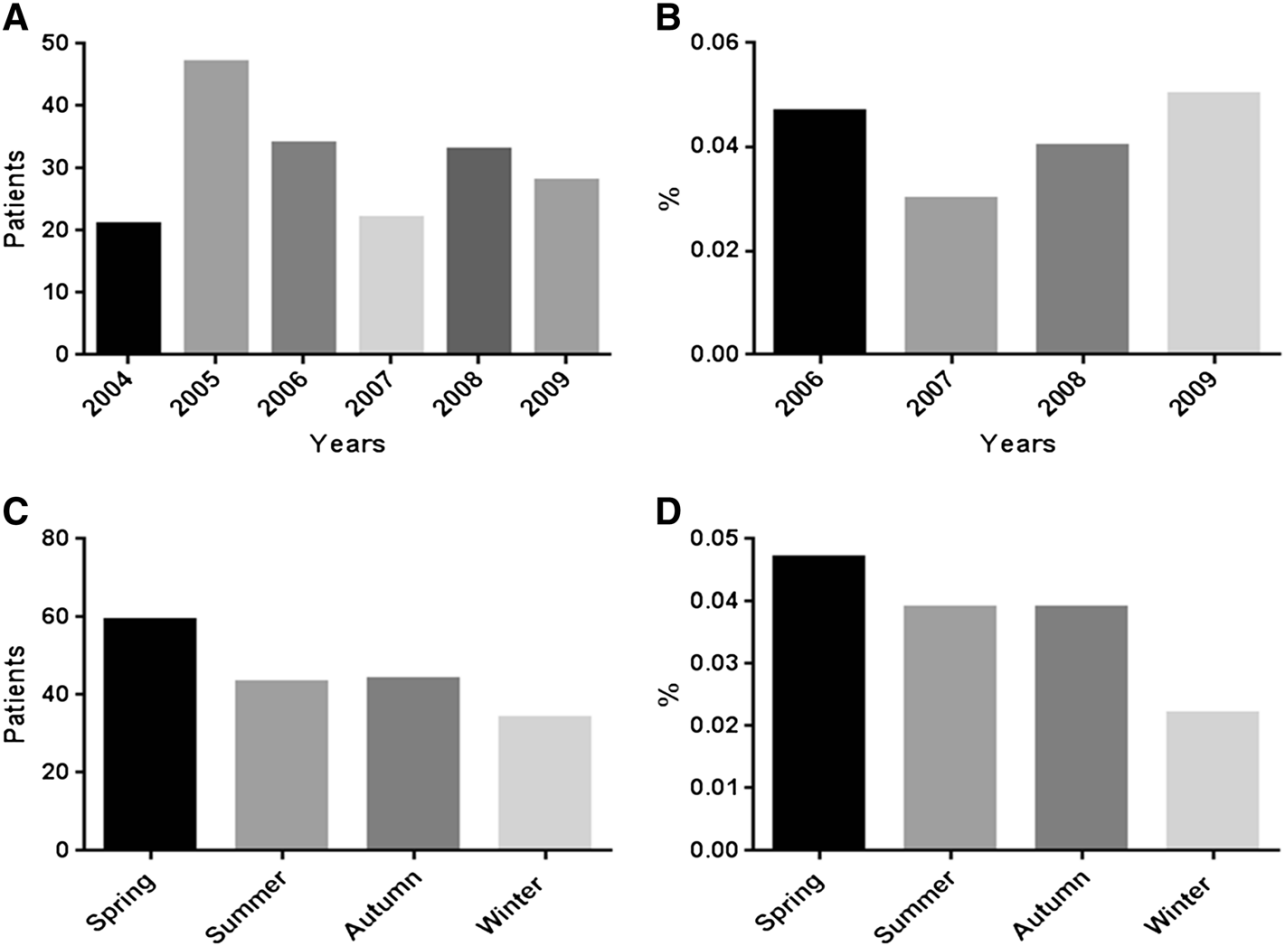
**Total number and incidence of infectious endophthalmitis and postoperative endophthalmitis in Gyungsang-do area, South Korea. A**. Total number of infectious endophthalmitis from 2004 to 2009 in Gyungsang-do area. Annual average number of infectious endophthalmitis was 30.0 cases/year (197 cases/6.5 year). **B**. Incidence rates of postoperative infectious endophthalmitis which was induced from division the number of postoperative infectious endophthalmitis by the number of cataract operation showed stepwise increase after 2006. **C**. Total number of infectious endophthalmitis from 2004 to 2009 stratified by season. The data of 2010 was excluded from the graphs **(A and** **B)** due to not enough data period. **D**. Incidence rate of postoperative infectious endophthalmitis stratified by season showed that spring was the highest season to develop the endophthalmitis.

A total of 197 patients were diagnosed and treated with infectious endophthalmitis. Aqueous humor or vitreous was cultured in 174 patients (88.3%), and 103 (59.2%) of them had positive results. Average presumed endophthalmitis incidence was 30.0 cases per year in Busan and Gyungsangnam-do area (Figure 1A). To evaluate the annual incident rate of postoperative infectious endophthalmitis, the numbers of cataract operation in 2006 ~ 2009 at the corresponding area were 40,693, 46,488, 49,545, and 56,021 based on KIRA database, and the corresponding number of postoperative endophthalmitis were 19, 14, 20, and 18. The corresponding incidence of postoperative endophthalmitis is 0.047%, 0.030%, 0.040%, and 0.049%, respectively. Overall, the incidence rate of postoperative infectious endophthalmitis over 4 years was 0.037% (Figure 1B).

The seasonal development of infectious endophthalmitis showed that spring appears the season in which infectious endophthalmitis developed most frequently (Figure 1C). To investigate the relationship between incidence of postoperative endophthalmitis and the seasonal variation, the number of cataract operation in the same area from 2006 to 2009 which was obtained from KIRA database and the number of postoperative infectious endophthalmitis was divided by the number of cataract operation, which showed that spring had the significantly highest incidence rate 0.047%, which is followed by summer 0.039%, autumn 0.039%, and winter 0.022% in the corresponding area during 2006 ~ 2009. (Figure 1D, p < 0.05, Chai-square test) In this regard, the seasonal variation was related with the incidence of postoperative endophthalmitis.

**Figure 2:**
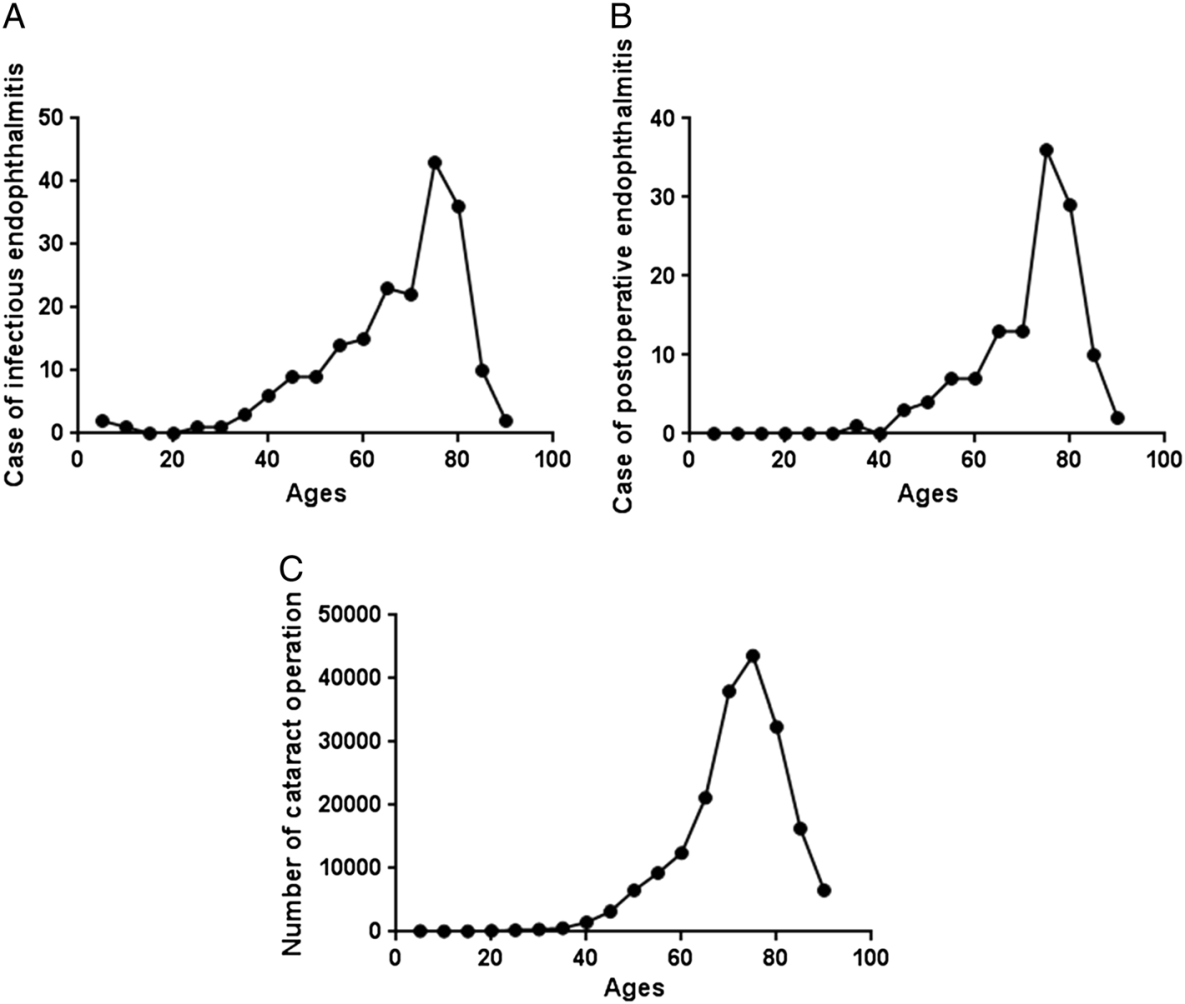
**Age distribution of infectious endophthalmitis in South Korea.** Average 66.9 ± 15.3 years.

Sex distribution was 49.8% men and 50.2% women. Laterality of eyes was right (54.8%), left (43.2%), and both (2.03%). Peak age of patients was eighth decade (36.6%), and mean age (standard deviation) was 66.9 (±15.3) years (Figure 2).

Past medical history was found in 48.2% of patients, and included hypertension (40.4%), diabetes (23.4%), liver disease (4.1%), cerebral infarction (3.5%), cardiac disease (2.9%), respiratory disease (2.3%), malignant tumor (2.3%), pulmonary tuberculosis (1.8%), kidney disease (1.8%), and others (4.7%). On the other hand, 35.6% of patients had no history of systemic diseases. No record of past medical history was found in 13.2% of patients.

**Figure 3:**
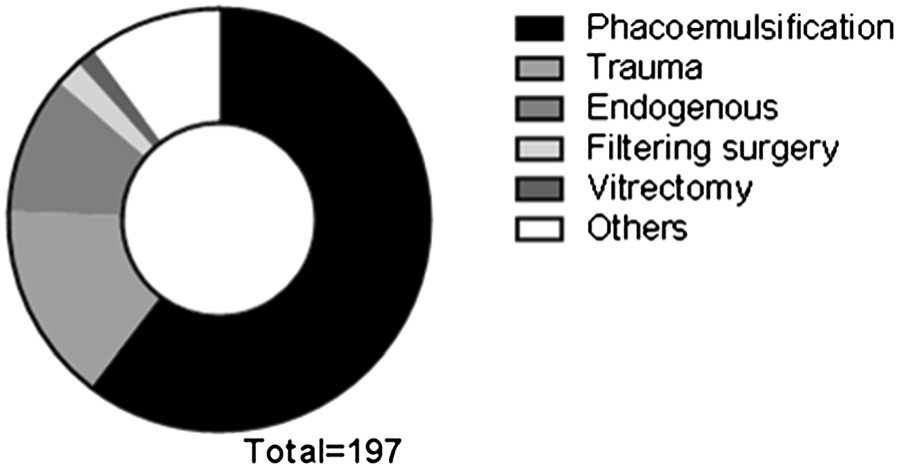
**Causes of infectious endophthalmitis in South Korea.**

The most common cause of infectious endophthalmitis was cataract operation (60.4%), followed by trauma (15.2%) and endogenous (10.7%). (Figure 3) Most of the cataract operations were uneventful phacoemulsifications (95.9%). Corneal laceration (51.6%) is the most common cause of traumatic endophthalmitis, followed by intraocular foreign body (25.8%), scleral and corneal laceration (9.7%), traumatic cataract (6.5%), and others (6.5%). In endogenous endophthalmitis, liver abscess (42.9%) is the most common related disease.

**Figure 4:**
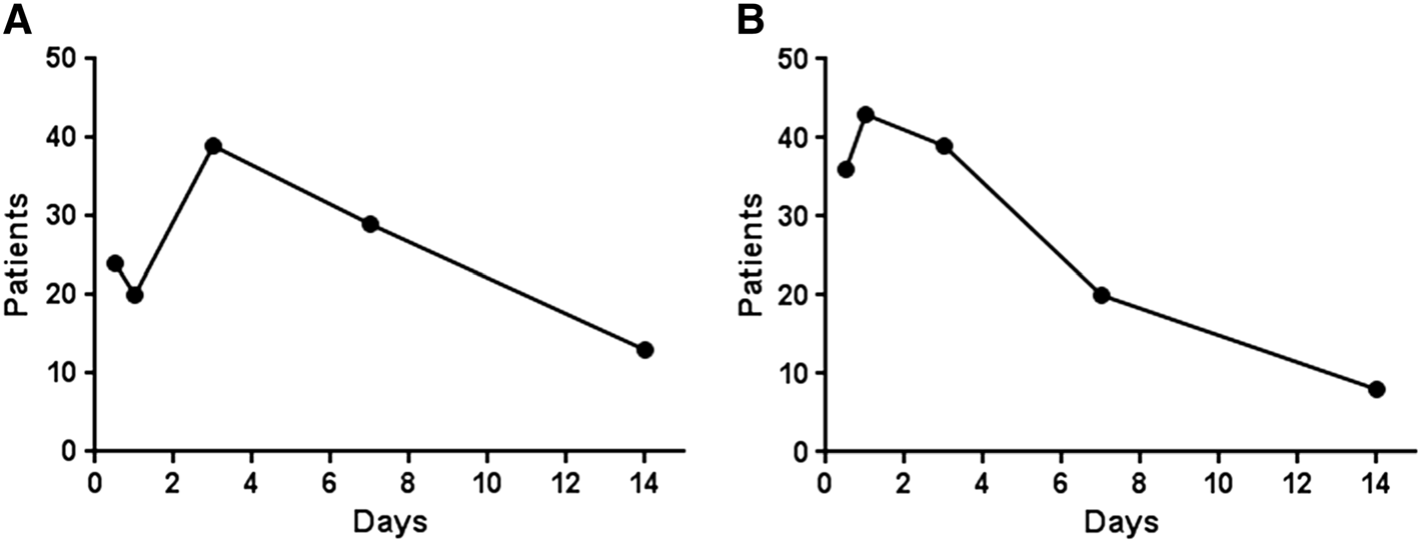
**Time interval between inciting event, symptom occurrence, and intervention in infectious endophthalmitis patients. A**. Duration of period from inciting event to symptom occurrence. The average duration was 29.8 ± 179.9 days. The number of cases in which the duration was over 14 days was 17 cases. **B**. Duration of period from symptom occurrence to intervention. The average duration was 3.9 ± 11.2 days. The number of cases in which the duration was over 14 days was 4 cases.

Most of the infectious endophthalmitis patients (85.5%) developed symptoms within two weeks after the event, and the peak period was 1–3 days (25.7%, Figure 4A). The average duration of the period from inciting event to symptom development was 29.8 (±179.9) days, while the duration of the period from symptom onset to initial intervention was 3.9 (±11.2) days (Figure 4B).

**Figure 5:**
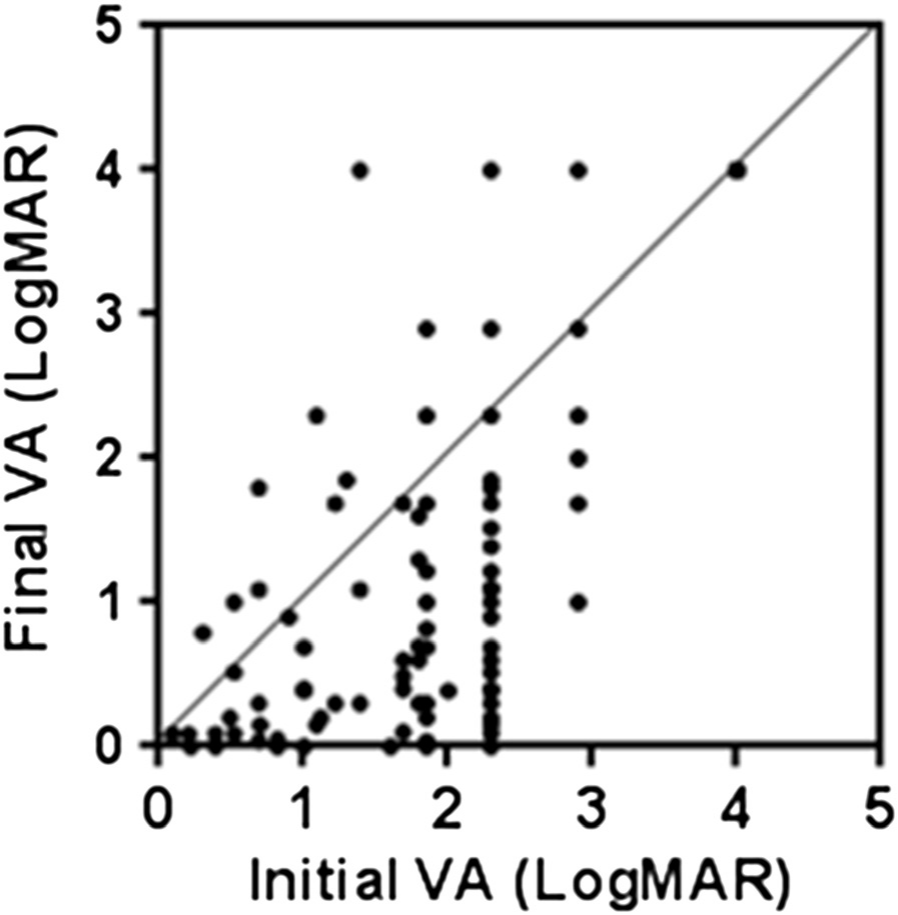
**Distribution of initial visual acuity (VA) and final VA in infectious endophthalmitis patients.** CF; Counting finger, LP; Light perception, HM; Hand movement, NLP; No light perception. All visual acuity examination was evaluated with Snellen chart.

The mean initial visual acuity of the patients was 2.01 (±0.70), and the mean final visual outcome was 1.46 (±1.04). The distributions of the initial and final visual outcomes are shown in Figure 5. About two-thirds of the patients had HM (hand movement) (44.4%) or less than HM (18.2%) during initial visual acuity testing; however, in the final visual outcome, about two-thirds of the patients had visual acuities more than counting fingers (62.2%). And the proportion of patients has final visual acuities above 0.2 was 33.3% (Figure 5).

As primary treatment for infectious endophthalmitis, combination of vitrectomy and intravitreal antibiotics injection simultaneously were chosen in 65.5% of patients (129/197), but intravitreal antibiotics injection only was used in 17.3% (34/197) (Table 1). The cases which were injected with intravitreal antibiotics first and then were converted to vitrectomy were 7.1% (14/197). Overall, the cases performed vitrectomy with or without intravitreal antibiotics injection were 72.6%. The most common antibiotics used as intravitreal injection agents were combination of vancomycin and ceftazidime (68.1%). Otherwise, the combination of vancomycin and amikacin was used 7.2% of the time. There were 7 cases of patients injected intravitreously with ceftazidime only. Quinolone topical antibiotics were used in 81.9%, among which the fourth-generation fluoroquinolone was used in 41.2%. Various antibiotics were used for systemic administration in infectious endophthalmitis. The systemic fourth-generation quinolone that can raise the intravitreal concentration most effectively in endophthalmitis patients was used in only 16.1% of patients in this study.

**Table 1:** **Infectious endophthalmitis treatment methods**

	**Number of cases**	**Percentage (%)**
Vitrectomy (with or without IAVI^1^)	143	72.6
Initial vitrectomy	129	65.5
Vitrectomy after IAVI	14	7.1
IAVI^1^ (with or without AC^2^ irrigation)	34	17.3
Others	20	10.2
Total	197	

^1^; Intravitreal antibiotics intravitreal injection.

^2^; Anterior chamber.

Vitrectomy was chosen as a primary intervention for infectious endophthalmitis in 129/197 cases (65.5%).

Overall positive culture rate in this study was 103/174 (59.2%). Positive culture rates varied depending on the culture method as follows: vitreous sampling before vitrectomy (50%), aqueous humor before intravitreal injection or vitrectomy (39.5%) and vitreous before intravitreal injection (37.5%). (Table 2) Most common microorganism which was cultured were *Enterococcus faecalis* 20.8% (20/96), which was followed by *Staphylococcus epidermidis* 18.8% (18/96) and Other Coagulase (−) staphylococcus 10.4% (10/96) [14].

**Table 2:** **Culture positivity depending on the sample harvesting method and the type of samples**

	**No. of samples**	**No. of culture positive samples**	**Culture positivity (%)**
Vitreous during PPV^1^	116	58	50.0
Aqueous humor	86	34	39.5
Vitreous during IVAI^2^	24	9	37.5
Blood culture	11	5	45.5
Others	9	5	55.6

^1^Pars plana vitrectomy.

^2^Intravitreal antibiotics injection.

Culture positivity of cases was 59.20% (103/174 cases).

## Discussion

Our literature search of infectious endophthalmitis studies in a Korean population included Korean Med (http://www.koreamed.org/), Pubmed (http://www.ncbi.nlm.nih.gov/), and the search engine of The Korean Ophthalmological Society (http://www.ophthalmology.org/) with term “infectious endophthalmitis”. This search revealed 11 articles, excepting case reports [7-10,15-20]. All papers were single-center studies and had limited numbers of patients or needed a long study period to recruit enough patients for analysis. Among the studies, the largest had 59 total cases and 37 culture-positive cases over the course of about 10 years. The smallest patient population had 18 total cases and 8 culture-positive cases over about 5 years [8,16]. In this context, the present study with its large study population of 174 total cases and 103 culture-positive cases has an advantage in revealing the clinical characteristics of Korean infectious endophthalmitis.

The annual incidence of postoperative infectious endophthalmitis was 0.037% in Busan and Gyeongsangnam-do. In the Swedish study, 0.029-0.048% of cataract operations developed postoperative endophthalmitis [6,21]. Recently, the incidence rate of infectious endophthalmitis in Canada was 0.14% of cataract operations [22]. A study in Singapore within the last 20 years revealed a rate of 0.076% [23]. The incident rate of South Korea was similar with the previous reports. It is worth to comment the stepwise increase of postoperative endophthalmitis rate during the period (2007 ~ 2009).

The seasonal variations of postoperative endophthalmitis were investigated. Most of previous studies showed no significant association between infectious endophthalmitis and season. However, seasonal differences in development of infectious endophthalmitis were observed in our study. Spring appears the season that develop infectious endophthalmitis most commonly. To clarify the seasonal variation of infectious endophthalmitis, the authors analyzed the seasonal incidence rate of postoperative infectious endophthalmitis over 4 years. Even though after adjusting for the number of cataract procedures performed in each season, the incident rate of spring is significantly higher than the others. On the other hand, a study performed in Australia showed that endophthalmitis occurred more often after operations performed in winter and autumn than after those performed in spring and summer [24]. But, the temperature of winter in Austrailia is similar to that of spring in South Korea. Rubio reported that conjunctival swab sample culture results had showed the frequency of bacteria had increased in April, May, and June, when the daily average temperature rises from 12°C to 22°C, this range is also similar to the temperature distribution of spring in South Korea [25]. In this regards, season or temperature could be associated with incidence of postoperative endophthalmitis.

The relationship between patient sex and infectious endophthalmitis is not clear throughout the previous studies [2,6,22,24]. Our study showed a similar distribution between sexes.

Right eyes had higher incidence of infectious endophthalmitis in our study. Wong and Chee’s Singapore data showed no significant difference [23].

Age over 85 years is a known risk factor for postoperative infectious endophthalmitis [6,21,22]. In our study, 1% (2/197) of infectious endophthalmitis patients was over 85 years. Most patients were between their 7^th^ and 8^th^ decades (59.4%).

Medical history of the patients was most frequently hypertension (40.4%) and diabetes (23.4%). These findings are generally in accordance with previous studies that found hypertension in 39.8% and diabetes in 13.8% [2,7,9]. Interestingly, 35.6% of the patients did not have any systemic problems when they developed infectious endophthalmitis. Lee and Park also reported that 54.2% of patients had no medical history [8].

Cataract surgery was the most common cause of infectious endophthalmitis, among which 95.9% of the operations were uneventful phacoemulsifications without anterior vitrectomy. Recent studies showed similar data [6,22].

In 84 of 152 cases (55.3%), signs of endophthalmitis occurred within 3 days after the inciting events, and 130 of 152 cases (85.5%) had signs of endophthalmitis within 14 days after the inciting events. This range was also in accordance with a previous study [26]. In 128 of 165 cases (77.6%), surgical interventions including pars plana vitrectomy, intravitreal antibiotics injection, or AC irrigation were performed within 3 days of endophthalmitis symptom onset.

The final visual outcome in this study was worse than previous studies [2,23,26]. Only 54 of 162 cases (33.3%) had final visual outcome over 20/100. EVS and Netherland studies showed that 74% and 68.8% of patients, respectively, achieved 20/100 or better vision. This difference could be explained by the included endophthalmitis categories and the microbiologic spectrum causing infectious endophthalmitis in our study. Traumatic and endogenous endophthalmitis groups were included in our study. *Enterococcus faecalis* was the most common causal bacteria in this study [14]. Recently, the visual outcome of Sweden’s study, in which the most common microorganism was *Enterococcus* species, was worse than EVS and Netherland, even though all of these studies included post-cataract operation endophthalmitis. In 40 of 135 cases (29.6%), the visual outcome was 20/40 or better [6]. Details about the factors related to the visual outcome are beyond this paper’s scope.

The rate of vitrectomy was 143 of 197 cases (72.6%), and the rate of only intravitreal antibiotic injection was 34 of 197 cases (17.3%). And the rate of vitrectomy as a primary treatment for infectious endophthalmitis was 65.5%. Even though the suggestion of the EVS study on postoperative endophthalmitis treatment, the surgeons who took part in this study preferred vitrectomy as the primary treatment rather than intravitreal antibiotic injection. The rate of vitrectomy as an early intervention in Netherland study was 25 of 250 cases (10%). However, the rate of vitrectomy in a recent Korean endophthalmitis study was 76.3%, which was similar to our current findings [8].

Culture positivity in our study was 103 of 174 cases (59.2%), which corresponded with the rates (54.4-69.3%) of the previous studies [23,26-29]. The higher positive culture rate in vitreous samples harvested during pars plana vitrectomy than in those harvested during intravitreal injection was quite interesting. This difference could be another advantage of vitrectomy as treatment for infectious endophthalmitis.

Infectious endophthalmitis could have different presentation, causative agents and therapeutic approaches depending on their different etiologies such as postoperative, traumatic, and endogenous endophthalmitis. Except the incidence of postoperative endophthalmitis, all kinds of infectious endophthalmitis were included and analyzed in this study. In this regards, the data in this study should be carefully interpreted and compared with the others results.

## Conclusions

This study of infectious endophthalmitis in South Korea over 5 years revealed several features showed different from the previous studies that visual outcome was worse, the vitrectomy was preferred for a primary treatment option for infectious endophthalmitis, seasonal variation was related with the development of postoperative infectious endophthalmitis. Moreover, it is also worthy to comment the increase of postoperative infectious endophthalmitis during 2007 ~ 2009 in our study. Further studies will be carried out for those differences and the cause of the increase of postoperative infectious endophthalmitis.

## Abbreviations

EVS: Endophthalmitis vitrectomy study
log-MAR: Log of the minimum angle of resolution
KIRA: Health Insurance Review and Assessment service of Korea
HM: Hand movement
